# Association between blood urea nitrogen concentration and diabetic foot ulcer: A retrospective cross-sectional study based on NHANES

**DOI:** 10.1097/MD.0000000000044038

**Published:** 2025-08-29

**Authors:** Ruijing Li, Changfan Li

**Affiliations:** a Guangzhou University of Chinese Medicine, Guangzhou, China; b The First Clinical College of Guangzhou University of Chinese Medicine, Guangzhou, China.

**Keywords:** blood urea nitrogen, cross-sectional, diabetic foot ulcer, NHANES

## Abstract

This study explores the relationship between blood urea nitrogen (BUN) levels and diabetic foot ulcer (DFU) in patients over the age of 40 in the United States. A total of 1673 participants who took part in the National Health and Nutrition Examination Survey (NHANES) from 1999 to 2004 were included in this study. Four logistic regression models were developed to assess the relationship between BUN levels and DFU. Smoothed curve-fitting analysis and threshold effect analysis were used to further specify the type of association. Subsequently, subgroup analyses were performed using stratified multiple regression analysis with age, sex, body mass index (BMI), and HbA1C. The study included 1622 adults with a mean age of 64.60 ± 11.82 years, of which 53.08% were male. Of the participants, 131 (8.07%) had DFU. In a multivariate logistic regression model, the highest BUN level was strongly associated with an increased risk of DFU (OR = 3.56, 95% CI: 1.47–8.62; *P* = .007). Smooth curve-fitting analysis showed a linear correlation between BUN and DFU (*P* for nonlinear = .056), with a threshold point of BUN = 5.4 mmol/L. These results suggest a possible linear positive correlation between BUN levels and DFU. Regular monitoring of BUN levels in diabetic patients can be helpful for early diagnosis and intervention of DFU.

## 1. Introduction

Diabetic foot ulcer (DFU) is a common complication among patients with type 2 diabetes mellitus. According to the International Diabetes Federation’s data from 2022, it is estimated that there are 537 million people with diabetes worldwide, and 19% to 34% of them will develop DFU at some point in their lives.^[1]^ Diabetes is the leading risk factor for lower limb amputations in adults in the United States, with an estimated 150,000 amputations related to diabetes occurring annually. The lifetime risk of lower limb amputation for patients with DFU is at least 19%.^[2]^ A recent meta-analysis involving 125,000 patients reported the following mortality rates for DFU: the 1-year mortality rate is 13.1%, the 5-year mortality rate is 49.1%, and the 10-year mortality rate is 76.9%. Cardiovascular diseases and infections are the main causes of death.^[3]^ The cost of each episode of wound care for advanced DFU exceeds $50,000, while the direct cost of major amputation surgery is even higher.^[4]^ The high incidence, amputation rate, and mortality rate of DFU impose a significant social and economic burden. Clarifying the pathological mechanisms of DFU and identifying susceptible populations is crucial for disease education and prevention.

Blood urea nitrogen (BUN) is a commonly used laboratory test in clinical practice, which measures the nitrogen content in blood urea. Elevated BUN levels are typically associated with renal insufficiency and may also be caused by prerenal factors such as congestive heart failure, severe burns and shock.^[5]^ In addition, some scholars have reported that BUN is also associated with various disease processes and outcomes, such as coronary artery disease,^[6]^ stroke,^[7]^ and sepsis.^[8]^

In recent years, studies have found that high levels of BUN are associated with the occurrence of diabetes and its complications. Xie et al conducted a follow-up study on over 1.3 million veterans and found that individuals with high BUN levels had a 23% higher risk of diabetes compared to those with normal BUN levels.^[9]^

Additionally, studies by Du and Luo^[10]^ and Xue and King^[11]^ based on the NHANES database have found a correlation between BUN levels and diabetes complications such as diabetic retinopathy and abdominal aortic calcification. Emerging evidence suggests that elevated BUN may not only reflect renal dysfunction but also contribute to systemic oxidative stress and insulin resistance, both of which are critical pathways in the pathogenesis of diabetic complications. For instance, experimental studies have demonstrated that urea accumulation directly impairs insulin secretion and promotes macrophage dysfunction, potentially exacerbating peripheral neuropathy and infection risks in diabetic patients.^[12,13]^ This mechanistic link provides a plausible rationale for investigating BUN as a biomarker for DFU.

While prior studies suggest associations between BUN and diabetes complications, only limited evidence exists for DFU specifically.^[14,15]^ The purpose of this study was to explore the relationship between BUN levels and patients with and without DFU using data from NHANES between 1999 and 2004.

## 2. Materials and methods

### 2.1. Data sources and study population

**Figure 1.**
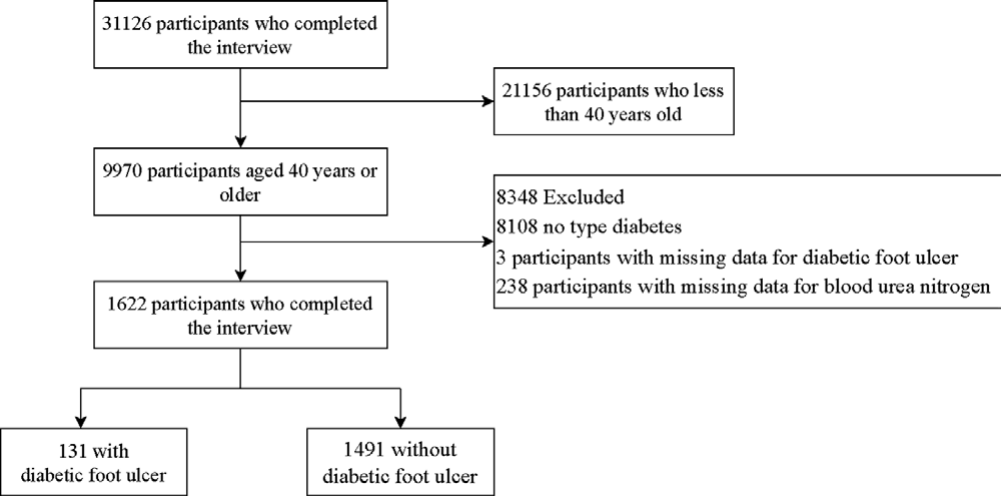
Flowchart of the participant selection. After excluding missing BUN and DFU information and others who did not meet the inclusion criteria, 1622 subjects were finally enrolled in this study. BUN = blood urea nitrogen, DFU = diabetic foot ulcer.

The National Health and Nutrition Examination Survey (NHANES) is one of the largest disease and health survey programs in the world, employing a multi-stage, complex stratified sampling method. It aims to assess the health and nutritional status of adults and children in the United States. The study was approved by the Ethics Review Board of the National Center for Health Statistics, and all participants provided written informed consent.^[16]^ The data used in this study is derived from the publicly accessible NHANES database (https://www.cdc.gov/nchs/nhanes/), hence no additional ethical approval or informed consent is required. This study is based on the survey data from NHANES between 1999 and 2004, selecting participants who were over 40 years old and completed both the questionnaire survey and the MEC screening. After excluding participants without diabetes (n = 8018) as well as those missing information on DFU (n = 3) and BUN data (n = 188), a total of 1673 participants were ultimately included in the study (Fig. 1). This study estimated the required sample size using the following formula: n = (*Z*α/2)^2 * *p* * (1 − *p*)/ δ^2. With a 95% confidence level, *Z*α/2 is 1.96, the estimated prevalence rate *p* is 0.2, and the margin of error δ is 2%. The calculated required sample size is 707 participants.^[17]^ Our study included 1624 participants, providing sufficient power (>80%) to detect the statistical association between BUN and the prevalence of DFU.

### 2.2. Determination of DFU

Type 2 diabetes mellitus is determined by the following criteria: 1. Elevated levels of glycated Hemoglobin A1c (HbA1c) exceeding or equal to 6.5%; 2. Fasting plasma glucose concentration greater than or equal to 7.0 mmol/L; 3. Blood glucose measurements taken 2 hours during an oral glucose tolerance test exceeding or equal to 11.1 mmol/L; 4. Self-reported physician diagnosis of diabetes; 5. Treatment with oral hypoglycemic agents or insulin.^[18]^ DFU is defined based on the patient’s response to the question in the problem data (DIQ090),“Have you had an ulcer or sore on your leg or foot that took more than 4 weeks to heal.”^[19]^

### 2.3. Determine BUN concentration

The BUN data were derived from blood samples collected from participants during the MEC screening. BUN samples were obtained from non-hemolyzed specimens and did not require fasting. If the samples are stored for more than 24 hours, they should be kept at a temperature between −15°C and −20°C, and are only allowed to be thawed once. Inadequate samples may include volumes that are insufficient (<0.6 mL), hemolysis, incorrect labeling, or serum or plasma being exposed to cells for too long. Urease is added to the sample, which hydrolyzes urea into carbon dioxide and ammonia. The generated ammonia reacts with α-ketoglutarate, NADH, and GLDH to produce glutamate and NAD+. The content of BUN in the sample is indirectly determined by measuring the change in absorbance caused by the consumption of NADH. The NHANES Laboratory/Medical Technicians Procedure Manual provides more detailed instructions for sample collection and processing.

### 2.4. Covariate

According to previous literature, this study includes the following factors as potential covariates for analysis: age, gender, race/ethnicity, education level, marital status, poverty income ratio (PIR), smoking status, alcohol consumption, body mass index (BMI), total cholesterol, C-reactive protein (CRP), and HbA1c.^[20,21]^ Additionally, a family history of diabetes, stroke, coronary heart disease, and hyperlipidemia, which are common comorbidities in DFU, have also been included in the analysis.^[10]^ Educational level is categorized into 3 groups: below high school, high school, and above high school. Marital status is divided into 2 categories: cohabiting and living alone.^[22]^ According to the PIR defined by the United States Census Bureau, family income is divided into 3 groups: low (PIR<1.3), medium (PIR 1.3 to 3.5), and high (PIR > 3.5). Alcohol consumption is categorized into 3 groups: never drinking, formerly drinking, and currently drinking. Smoking status is categorized into 3 groups: never smokers, former smokers, and current smokers.^[23]^ HbA1c levels are divided into 2 categories: <6.5% or 6.5% and above. The history of previous diseases such as hypertension, stroke, and hyperlipidemia is obtained from questionnaires that ask: “Have you ever been told by a medical professional that you have this condition?”

### 2.5. Statistical analysis

The NHANES survey employed a complex, multi-stage, probability sampling method. To obtain research results that more closely approximate a national sample, this study combined sample weights from multiple cycles for weighted analysis. All statistical analyses were performed using the R statistical software(Version 4.2.2, http://www.R-project.org, The R Foundation). Categorical data were described using frequency (n) and percentage (%), while continuous data were described using mean ± standard deviation (SD). For continuous variables, independent sample *t*-tests were used, and for categorical variables, chi-square (χ^2^) tests were used to compare baseline characteristics. This study categorized the concentration of BUN and the study participants into 4 groups (Quartile 1–Quartile 4) based on the quartiles of BUN. For the BUN quartiles, the general characteristics were compared using the Wilcoxon rank-sum test for continuous variables and the chi-square test for categorical variables. Four separate multiple logistic regression models were used to examine the independent association between BUN and DFU. The crude model did not adjust for confounding variables. Model 1 included age, gender, race, education level, household income, and marital status. Model 2, building on Model 1, added BMI, cardiovascular diseases, stroke, family history of diabetes, hyperlipidemia, alcohol consumption, and smoking status. Model 3 included all the factors from Model 2, along with additional factors such as HbA1c, CRP, and total cholesterol. Subgroup analyses were performed using stratified multiple regression analysis with age, gender, BMI, and HbA1c as the variables of interest to explore the association between BUN and DFU. Additionally, an interaction term was introduced to check for heterogeneity in the associations between subgroups. Then, smooth curve analysis was used to determine whether there is a linear correlation between BUN and DFU, and threshold effect analysis was conducted on the inflection points obtained from the smooth curve-fitting analysis.

## 3. Result

### 3.1. Baseline characteristics

This study included a total of 1622 diabetic participants over the age of 40, with an average age of 64.60 ± 11.82 years, of which 53.08% were male. Among the participants, 131 had DFU, accounting for 8.07%. Table 1 presents the baseline characteristics of study participants classified by BUN quartiles. Significant differences were observed across the 4 BUN quartile groups in terms of gender, age, race, marital status, smoking status, coronary heart disease, hypertension, glycated hemoglobin, stroke, and hemoglobin. Table 2 categorizes participants into those with DFU and those without DFU. There are significant differences in BUN levels, hemoglobin levels, marital status, and cardiovascular disease status between the 2 groups (*P* < .05).

**Table 1:** Weighted characteristics of participants based on BUN level quartiles.

Characteristics	BUN (blood urea nitrogen)				
	Quartile 1 <4.28	Quartile 2 (4.28,5.36)	Quartile 3 (5.36,6.78)	Quartile 4 >6.48	*P*-value
	n = 354	n = 343	n = 400	n = 525	
Sex, n (%)					
Male	151 (42.7)	178 (51.9)	232 (58.0)	300 (57.1)	<.001
Female	203 (57.3)	165 (48.1)	168 (42.0)	225 (42.9)	
Age (yr), mean (SD)	59.42 ± 11.46	61.89 ± 11.38	65.14 ± 11.44	69.44 ± 10.62	<.001
Race/ethnicity, n (%)					
Mexican American	112 (31.6)	105 (30.6)	115 (28.7)	134 (25.5)	<.001
Non-Hispanic White	105 (29.7)	130 (37.9)	163 (40.8)	260 (49.5)	
Non-Hispanic Black	106 (29.9)	81 (23.6)	87 (21.8)	95 (18.1)	
Other	31 (8.8)	27 (7.9)	35 (8.8)	36 (6.9)	
Education level, n (%)					
above high school	116 (32.8)	100 (29.2)	129 (32.2)	157 (29.9)	.8
below high school	165 (46.6)	175 (51.0)	186 (46.5)	266 (50.7)	
high school	73 (20.6)	68 (19.8)	85 (21.2)	102 (19.4)	
Marital, n(%)					
married or living with partners	188 (53.1)	224 (65.3)	242 (60.5)	292 (55.6)	.004
alone	166 (46.9)	119 (34.7)	158 (39.5)	233 (44.4)	
PIR, n (%)					
low	163 (46.0)	137 (39.9)	152 (38.0)	211 (40.2)	.324
medium	127 (35.9)	126 (36.7)	156 (39.0)	204 (38.9)	
high	64 (18.1)	80 (23.3)	92 (23.0)	110 (21.0)	
Hypertension (%)					
Yes	198 (55.9)	215 (62.7)	248 (62.0)	373 (71.0)	<.001
No	156 (44.1)	128 (37.3)	152 (38.0)	152 (29.0)	
Hyperlipidemia (%)					
Yes	154 (43.5)	164 (47.8)	201 (50.2)	255 (48.6)	.295
No	200 (56.5)	179 (52.2)	199 (49.8)	270 (51.4)	
Cardiovascular disease, n (%)					
Yes	26 (7.3)	29 (8.5)	43 (10.8)	89 (17.0)	<.001
No	328 (92.7)	314 (91.5)	357 (89.2)	436 (83.0)	
Stroke, n (%)					
Yes	27 (7.6)	18 (5.2)	32 (8.0)	63 (12.0)	.004
No	327 (92.4)	325 (94.8)	368 (92.0)	462 (88.0)	
Alcohol status, n (%)					
never	62 (18.8)	63 (19.0)	73 (19.1)	98 (20.1)	.997
former	246 (74.8)	249 (75.0)	288 (75.4)	360 (73.8)	
current	21 (6.4)	20 (6.0)	21 (5.5)	30 (6.1)	
Smoking status, n (%)					
never	158 (44.6)	161 (47.2)	189 (47.2)	233 (44.5)	<.001
former	106 (29.9)	127 (37.2)	155 (38.8)	228 (43.5)	
current	90 (25.4)	53 (15.5)	56 (14.0)	63 (12.0)	
Diabetes family history, n (%)					
Yes	259 (73.2)	237 (69.1)	272 (68.0)	346 (65.9)	.151
No	95 (26.8)	106 (30.9)	128 (32.0)	179 (34.1)	
BMI (kg/m^2^), mean (SD)	31.62 ± 6.82	31.32 ± 6.26	30.86 ± 6.99	30.59 ± 6.24	.122
CRP (mg/L), median (IQR)	0.86 (0.75)	0.60 (0.57)	0.66 (0.57)	0.75 (0.54)	.101
HbA1c (%), mean (SD)	7.55 ± 1.84	7.54 ± 1.97	7.48 ± 1.79	7.25 ± 1.67	.04
Total cholesterol, mean (SD)	5.38 ± 1.30	5.21 ± 1.15	5.18 ± 1.07	5.20 ± 1.24	.079
Hemoglobin (g/L), mean (SD)	14.30 ± 1.57	14.43 ± 1.51	14.29 ± 1.52	13.66 ± 1.65	<.001
Diabetic foot ulcer, n (%)					
Without DFU	328 (92.7)	330 (96.2)	380 (95.0)	453 (86.3)	<.001
DFU	26 (7.3)	13 (3.8)	20 (5.0)	72 (13.7)	

Mean ± SD was for continuous variables: the *P*-value was calculated by the linear regression model. Median [IQR] for skewed continuous variables. n % was for categorical variables: the *P*-value was calculated by the chi-square test.

BMI = body mass index, BUN = blood urea nitrogen, CRP = C-reactive protein, DFU = diabetic foot ulcer, HbA1c = glycosylated hemoglobin, IQR = interquartile range, PIR = poverty income ratio.

**Table 2:** Weighted characteristics of participants based on diabetic foot ulcer calcification.

	Without DFU, n = 1491 (91.9%)	DFU, n = 131 (8.1%)	*P*-value
Sex, n (%)			
Male	784 (52.6)	77 (58.8)	.204
Female	707 (47.4)	54 (41.2)	
Age (yr), mean (SD)	64.56 ± 11.80	64.98 ± 12.05	.695
Race/ethnicity, n (%)			
Mexican American	426 (28.6)	40 (30.5)	.811
Non-Hispanic White	603 (40.4)	55 (42.0)	
Non-Hispanic Black	341 (22.9)	28 (21.4)	
Other	121 (8.1)	8 (6.1)	
Education level, n (%)			
Above high school	456 (30.6)	46 (35.1)	.548
Below high school	731 (49.0)	61 (46.6)	
High school	304 (20.4)	24 (18.3)	
Marital, n (%)			
Married or living with partners	881 (59.1)	65 (49.6)	.044
Alone	610 (40.9)	66 (50.4)	
PIR, n (%)			
Low	601 (40.3)	62 (47.3)	.286
Medium	568 (38.1)	45 (34.4)	
High	322 (21.6)	24 (18.3)	
Hypertension (%)			
Yes	947 (63.5)	87 (66.4)	.571
No	544 (36.5)	44 (33.6)	
Hyperlipidemia (%)			
Yes	707 (47.4)	67 (51.1)	.467
No	784 (52.6)	64 (48.9)	
Cardiovascular disease, n (%)			
Yes	164 (11.0)	23 (17.6)	.035
No	1327 (89.0)	108 (82.4)	
Stroke, n (%)			
Yes	129 (8.7)	11 (8.4)	1
No	1362 (91.3)	120 (91.6)	
Alcohol status, n (%)			
Never	274 (19.4)	22 (18.2)	.812
Former	1050 (74.5)	93 (76.9)	
Current	86 (6.1)	6 (5.0)	
Smoking status, n (%)			
Never	684 (46.0)	57 (43.5)	.493
Former	568 (38.2)	48 (36.6)	
Current	236 (15.9)	26 (19.8)	
Diabetes family history, n (%)			
Yes	1015 (68.1)	99 (75.6)	.094
No	476 (31.9)	32 (24.4)	
BMI (kg/m^2^), mean (SD)	30.95 ± 6.47	32.20 ± 7.72	.051
CRP (mg/L), median (IQR)	0.71 (0.58)	0.85 (0.83)	.314
HbA1c (%), mean (SD)	7.42 ± 1.78	7.58 ± 2.10	.341
Total cholesterol, mean (SD)	5.25 ± 1.20	5.10 ± 1.19	.169
Hemoglobin (g/L), mean (SD)	14.16 ± 1.59	13.66 ± 1.64	.001
BUN (mmol/L), mean (SD)	6.09 ± 3.18	7.79 ± 4.25	<.001

Mean ± SD was for continuous variables: the *P*-value was calculated by the linear regression model. Median [IQR] for skewed continuous variables. n % was for categorical variables: the *P*-value was calculated by the chi-square test.

BMI = body mass index, BUN = blood urea nitrogen, CRP = C-reactive protein, DFU = diabetic foot ulcer, HbA1c = glycosylated hemoglobin, PIR = poverty income ratio.

### 3.2. Factors associated with DFU

Univariate ordinal regression analysis showed that marital status, cardiovascular disease, and hemoglobin levels are associated with DFU (*P* < .05; Table 3).

**Table 3:** Univariate analysis for the presence of diabetic foot ulcer.

Characteristic	OR (95% CI)	*P*-value
BUN	1.11 (1.06–1.15)	<.001
Sex, n (%)		
Male	1	
Female	0.78 (0.54–1.12)	.174
Age(year)	1.00 (0.99–1.02)	.695
Race/ethnicity, n (%)		
Mexican American	1	
Non-Hispanic Black	0.87 (0.53–1.45)	.602
Non-Hispanic White	0.97 (0.63–1.49)	.894
Other	0.70 (0.32–1.54)	.381
Education level, n (%)		
Below high school	1	
High school	0.95 (0.58–1.55)	.825
Above high school	1.21 (0.81–1.80)	.353
Marital, n (%)		
Alone	1	
Married or living with partners	0.68 (0.48–0.98)	.036
PIR, n (%)		
Low	1	
Medium	0.77 (0.51–1.15)	.196
High	0.72 (0.44–1.18)	.194
Alcohol status, n (%)		
Never	1	
Former	1.10 (0.68–1.79)	.691
Current	0.87 (0.34–2.21)	.768
Smoking status, n (%)		
Never	1	
Former	1.01 (0.68–1.51)	.945
Current	1.32 (0.81–2.15)	.261
Hypertension, n (%)		
No	1	
Yes	1.14 (0.78–1.66)	.509
Hyperlipidemia, n (%)		
No	1	
Yes	1.16 (0.81–1.66)	.413
Stroke, n (%)		
No	1	
Yes	0.97 (0.51–1.84)	.921
Cardiovascular disease, n (%)		
No	1	
Yes	1.72 (1.07–2.78)	.026
Family history of diabetes, n (%)		
No	1	
Yes	1.45 (0.96–2.19)	.077
BMI (kg/m^2^)	1.03 (1.00–1.05)	.051
CRP (mg/L)	1.05 (0.96–1.15)	.323
HbA1c (%)	1.05 (0.95–1.15)	.341
Hemoglobin (g/L)	0.83 (0.74–0.92)	<.001
Total cholesterol (mg/dL)	0.89 (0.76–1.05)	.168

n % was for categorical variables: the *P*-value was calculated by the univariate analysis.

BMI = body mass index, BUN = blood urea nitrogen, CRP = C-reactive protein, DFU = diabetic foot ulcer, HbA1c = glycosylated hemoglobin, PIR = poverty income ratio.

### 3.3. Relationship between BUN and DFU

The association between BUN and DFU was identified using 4 logistic regression models, with the results presented as odds ratios (OR) and their 95% confidence intervals (CI) in Table 4. When BUN was analyzed as a continuous variable, the unadjusted model (OR = 1.13, 95% CI: 1.06–1.21; *P* < .001) and the 3 adjusted models consistently showed a significant positive association between BUN and DFU (Model 3: OR = 1.11, 95% CI: 1.02–1.21; *P* = .017). When BUN was analyzed as a categorical variable based on quartile intervals (Quartile 1–Quartile 4), with the lowest quartile Quartile 1 set as the reference category (dummy variable). All 4 models consistently showed that in Quartile 4, DFU exhibited a higher incidence rate (crude model: OR = 2.59, 95% CI: 1.31–5.11; *P* = .007; Model 1: OR = 3.15, 95% CI: 1.50–6.64; *P* = .004; Model 2: OR = 4.13, 95% CI: 1.77–9.62; *P* = .002; Model 3: OR = 3.56, 95% CI: 1.47–8.62; *P* = .007; all *P* for trend < .05).

**Table 4:** Multivariate logistic regression models of blood urea nitrogen with diabetic foot ulcer.

	Crude Model		Model 1		Model 2		Model 3	
	OR (95% CI)	*P*-value	OR (95% CI)	*P*-value	OR (95% CI)	*P*-value	OR (95% CI)	*P*-value
BUN (continuous)	(1.06–1.21)	<.001	(1.07–1.24)	<.001	(1.05–1.24)	.003	(1.02–1.21)	.017
BUN (quartile)								
Quartile 1	Reference		Reference		Reference		Reference	
Quartile 2	(0.16–1.68)	.267	(0.17–1.91)	.351	(0.23–3.22)	.828	(0.22–3.22)	.781
Quartile 3	(0.36–2.49)	.92	(0.39–2.93)	.905	(0.47–4.20)	.526	(0.43–3.91)	.625
Quartile 4	(1.31–5.11)	.007	(1.50–6.64)	.004	(1.77–9.62)	.002	(1.47–8.62)	.007
*P* for trend	(1.19–2.00)	.002	(1.24–2.16)	<.001	(1.33–2.32)	<.001	(1.23–2.25)	.002

Crude model: unadjusted model; Model 1: adjusted for sociodemographic variables (age, sex, race, marital status, education level, PIR); Model 2: Model 1 and BMI, hypertension, cardiovascular disease, stroke, family history of diabetes, hyperlipidemia, alcohol status, smoking status; Model 3: adjusted for Model 2, HbA1c, total cholesterol, hemoglobin, CRP.

BMI = body mass index, CRP = C-reactive protein, DFU = diabetic foot ulcer, HbA1c = glycosylated hemoglobin, OR = odds ratio, PIR = poverty income ratio.

**Figure 2.**
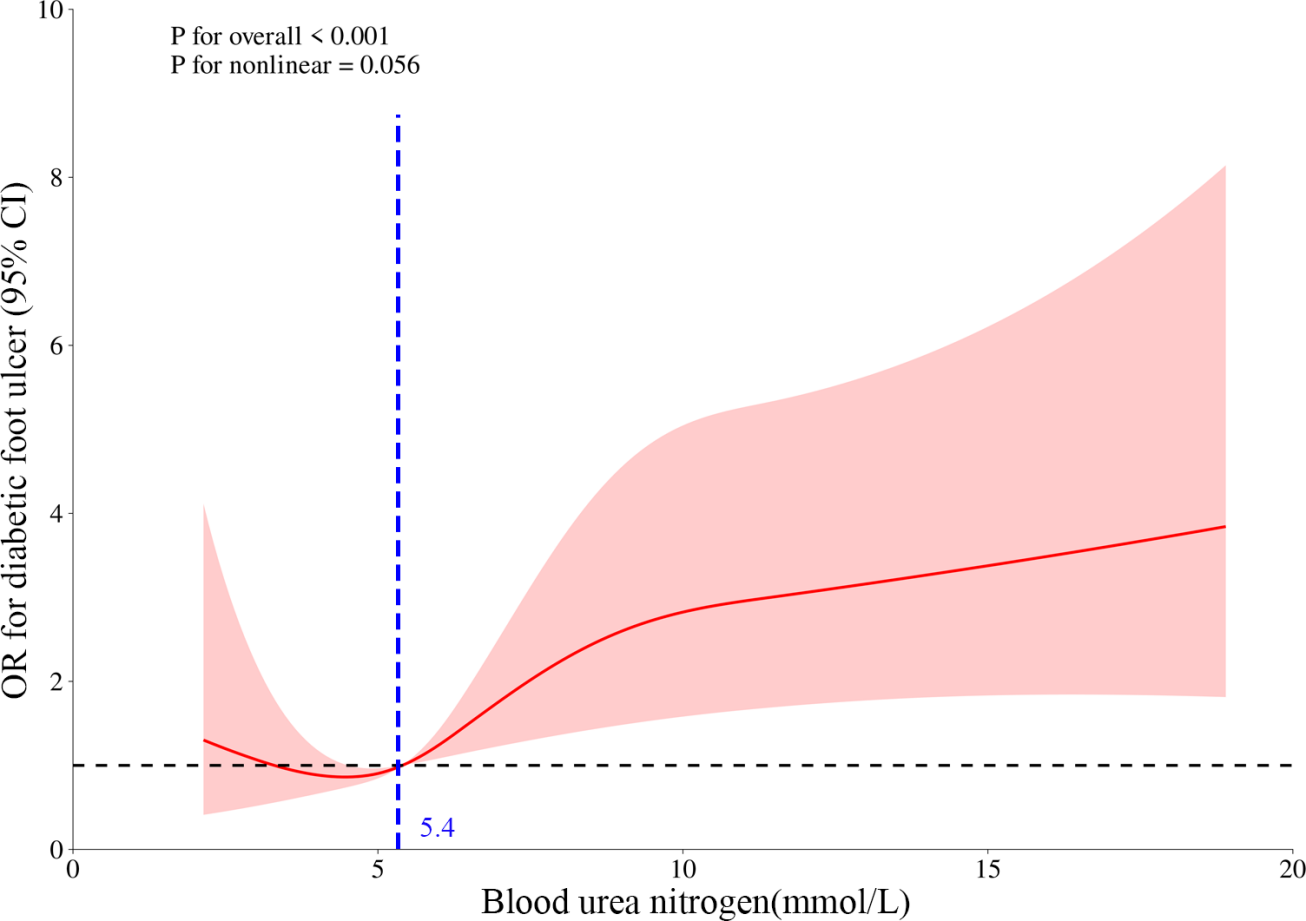
Association between BUN level and diabetic foot ulcer. After adjusting for covariates such as age, sex, race, marital status, and education level, the smoothed curve analysis showed a linear correlation between BUN concentration and the risk of developing DFU, with a risk BUN cutoff of 5.4 mmol/L. BUN = blood urea nitrogen, DFU = diabetic foot ulcer.

In the fully adjusted model (Model3), the smooth curve analysis showed a linear correlation between BUN and DFU (Fig. 2). Subsequently, a threshold effect analysis was conducted on the association between BUN and DFU, fitting the models as a standard linear regression model and a 2-piecewise linear regression model. The results indicated that there was no difference in efficacy between the standard linear regression model and the 2-piecewise linear regression model (likelihood ratio test *P*-value > .05), and the inflection point of BUN was identified as 5.4 mmol/L (Table 5). When BUN is ≥ 5.4 mmol/L, each doubling of BUN is significantly associated with a 7.2% increase in the prevalence of DFU (OR = 1.072, 95% CI: 1.013–1.133; *P* = .014). However, when BUN is < 5.4 mmol/L, the relationship between BUN and DFU is not statistically significant (OR = 1.262, 95% CI: 0.948–1.729; *P* = .128).

**Table 5:** Threshold effect analysis of blood urea nitrogen with diabetic foot ulcer using standard linear regression model.

BUN	Adjust OR (95% CI)	*P*-value
≤5.4 mmol/L	1.26 (0.95–1.72)	.128
>5.4 mmol/L	1.07 (1.01–1.13)	.014
Likelihood Ratio test	–	.303

Adjusted for age, sex, race, marital status, education level, PIR, BMI, hypertension, cardiovascular disease, stroke, family history of diabetes, hyperlipidemia, alcohol status, smoking status, HbA1c, total cholesterol, hemoglobin and CRP.

BMI = body mass index, CRP = C-reactive protein, HbA1c = glycosylated hemoglobin, OR = odds ratio, PIR = poverty income ratio.

### 3.4. Subgroup Analyses of Factors Influencing the Association Between BUN and the Presence of DFU

**Figure 3.**
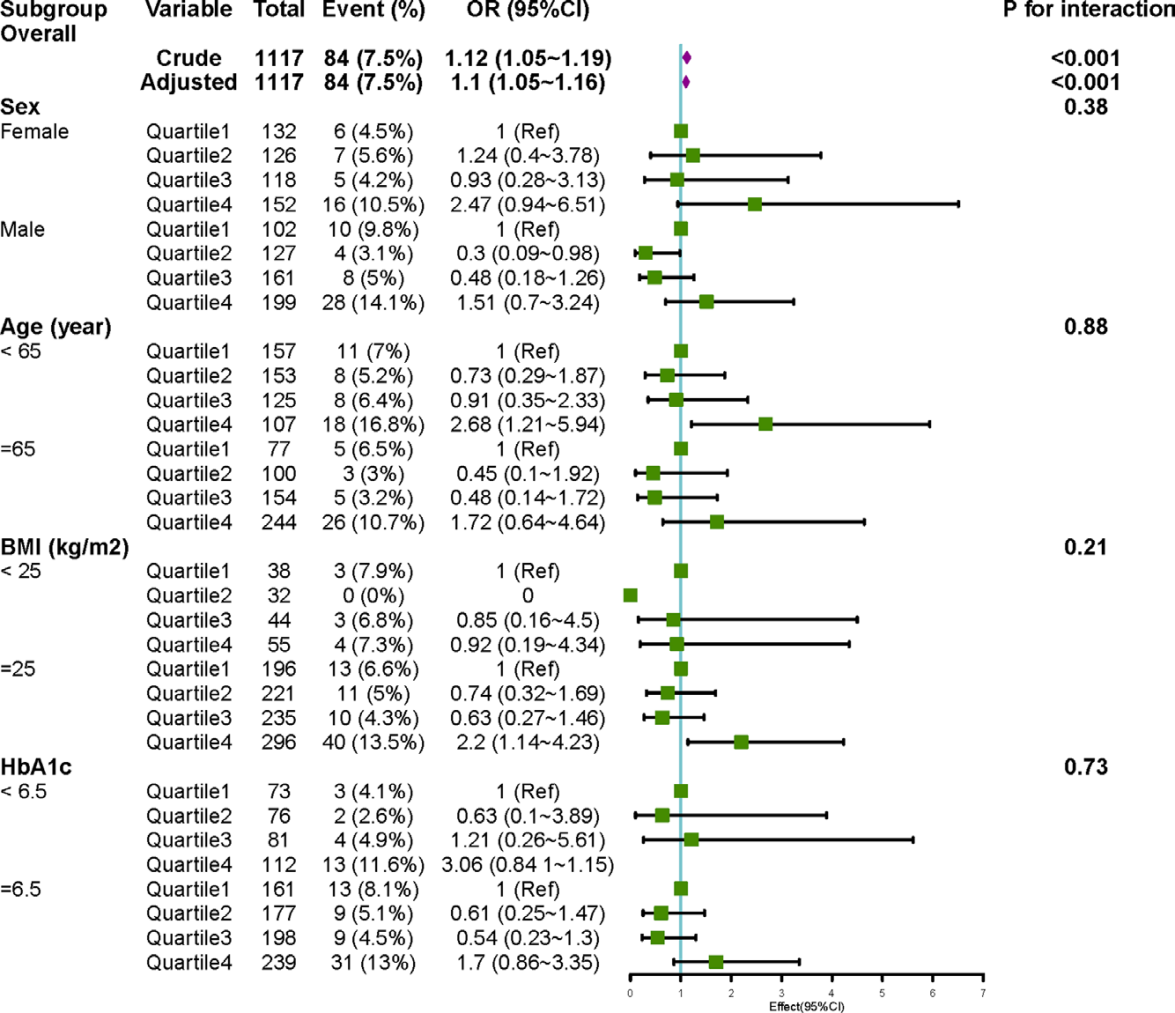
Subgroup analysis of BUN levels on the presence of diabetic foot ulcer. No significant result bias was observed in the subgroup analyses of sex, age, BMI, and HbA1c, suggesting that the effect of BUN on DFU was stable. BMI = body mass index, BUN = blood urea nitrogen, DFU = diabetic foot ulcer, HbA1c = glycated hemoglobin A1c.

Subgroup analyses were conducted with age, gender, BMI, and HbA1c as stratification variables, and the results are shown in Figure 3. After stratification by gender, age, HbA1c levels, or BMI, no significant interactions were observed in any subgroup (all *P*-values for interaction > .05).

## 4. Discussion

This study conducted a retrospective analysis of the diabetic population in the NHANES database from 1999 to 2004 and found that BUN levels had a significantly positive correlation with the risk of developing DFU. The initial sample size estimation (n = 707) was based on an anticipated DFU prevalence of 20%, derived from prior literature.^[17]^ However, the observed prevalence in our cohort was lower (8.07%). Despite this discrepancy, post hoc power analysis confirmed that the final sample (n = 1622) provided > 80% power to detect an OR of 3.56 for the highest BUN quartile, assuming a 2-tailed α = 0.05. Smooth curve analysis revealed a linear correlation between BUN concentration and DFU risk, with a threshold point at 5.4 mmol/L. And the threshold approximates the upper limit of normal renal function (reference range: 2.5–7.1 mmol/L), suggesting that even subclinical elevations in BUN may confer DFU risk. This aligns with CKD staging criteria, where early renal impairment (Stage 2, eGFR 60–89 mL/min/1.73m²) is associated with subtle BUN increases. The lack of significant interactions in subgroup analyses, such as age, sex, HbA1c, implies that the BUN-DFU association is robust across diverse diabetic populations. This universality underscores BUN’s potential as a broadly applicable screening tool, independent of conventional risk stratification factors. The study results suggest that diabetic patients with higher BUN levels should have regular monitoring, early intervention, and active prevention to avoid the occurrence of DFU. This reveals the potential of BUN as a predictive biochemical marker for DFU and contributes to the prevention, treatment, and prognosis assessment of the disease. To our knowledge, this study is the first cross-sectional study based on the NHANES database to explore the relationship between BUN and the risk of DFU in the American diabetic population.

Some early studies have indicated a correlation between BUN levels and DFU. Jiang et al conducted a retrospective cohort study involving 1333 diabetic patients, and the results showed a significant difference in BUN levels between patients with DFU and those without DFU (*P* < .0001).^[24]^ Wang et al conducted a retrospective study on 1333 patients with DFU.^[25]^ The results of the multivariate logistic regression indicated that BUN is a risk factor for the recurrence of DFU (OR = 1.06, 95% CI: 1.01–1.12; *P* < .05). Additionally, the receiver operating characteristic curve analysis in both the derivation and validation cohorts showed that the regression model including BUN had the best predictive performance for the recurrence of DFU. These findings are largely consistent with our results.

Oxidative stress plays a pivotal role in the pathogenesis of diabetic complications, directly damaging vascular endothelium and impairing tissue repair mechanisms. It involves a variety of biological processes and molecular mechanisms, which can directly damage vascular endothelial cells, affect the normal function of blood vessels, and subsequently lead to vasculitis, thrombosis, and other conditions.^[26]^ As early as 1982, the presence of oxidative phenomena in the bodies of diabetic patients was discovered.^[27]^

The abnormally high blood sugar levels in diabetic patients can lead to the overproduction of various reactive oxygen species (ROS). These ROS can cause cellular and tissue damage through different signaling pathways, ultimately contributing to the development of various diabetic complications.^[28]^ ROS can cause vascular damage and remodeling through mechanisms such as inhibiting the expression of sestrin proteins, altering the direction of action of uncoupling protein 2, and stimulating the platelet-derived growth factor signaling. Microvascular damage and occlusion are significant causes of diabetic retinopathy, diabetic nephropathy, and DFU.^[29–32]^ Therefore, we speculate that BUN not only reflects the status of kidney function but also to some extent reflects the degree of injury to extrarenal microvessels.

Some studies have found a close link between blood urea/BUN levels and insulin resistance.^[33,34]^ Animal experiments by Koppe et al showed that elevated circulating urea levels increase O-GlcNAcylation of islet proteins and impair glycolysis, ultimately leading to impaired insulin secretion function in mice.^[12]^ D’Apolito et al’s research found that cultured adipocytes treated with urea exhibited reduced insulin sensitivity. In a mouse model of renal failure, uremic mice displayed insulin resistance and glucose intolerance, and normal mice infused with urea also developed the same degree of insulin resistance.^[13]^

Peripheral vascular disease, peripheral neuropathy, and infection are 3 important factors in the occurrence of DFU.^[35]^ Insulin resistance increases the difficulty of blood glucose control in diabetic patients. Long-term hyperglycemia can lead to increased activity of the polyol pathway, formation of advanced glycation end products, activation of protein kinase C, and abnormalities in various other metabolic pathways. These changes subsequently affect the survival of nerve cells and the repair and regeneration of nerve fibers, ultimately leading to neurological dysfunction.^[36]^ Insulin resistance can also affect the metabolism and function of immune cells such as T lymphocytes and macrophages, leading to a decline in the body’s immune defense capabilities and increasing the risk of infection. Tsai et al found that in the H1N1-infected insulin receptor-deficient (INSR-deficient) mouse model, the absolute number or percentage of influenza-specific CD4+ T cells in the lungs and draining lymph nodes on day 9 postinfection were significantly reduced in INSR-deficient mice compared to non-deficient mice.^[37]^ Ieronymaki et al discovered that in a mouse model of sepsis induced by cecal ligation and puncture, mice with insulin-resistant macrophages had a higher bacterial load in the peritoneal lavage at the same time points compared to normal mice.^[38]^ Our findings align with preclinical evidence showing that urea-induced oxidative stress disrupts vascular endothelial function and accelerates neuropathy, which are key contributors to DFU development. Specifically, elevated BUN may impair nitric oxide bioavailability and promote advanced glycation end product formation, leading to microvascular occlusion and delayed wound healing.^[29,31]^

However, this study has some limitations. Firstly, as it is a retrospective study, it is not possible to eliminate the bias that missing data may introduce to the results. Secondly, due to changes in the DFU survey questionnaire within the NHANES database, we only utilized survey data from 1999 to 2004, as they provided a more accurate definition of patients with DFU. However, the reliance on self-reported DFU (NHANES item DIQ090) may introduce misclassification bias, as nondiabetic ulcers or minor wounds could be erroneously included. Future prospective studies incorporating clinical examinations are warranted to validate our findings. Additionally, the use of NHANES data from 1999–2004 may limit generalizability to contemporary populations, given advancements in diabetes management. Third, the temporal relationship between BUN and DFU cannot be ascertained, leaving open the possibility of reverse causation. Fourth, due to limitations in the data sources, it was not possible to incorporate the severity grading of DFU, and BUN was measured at a single time point, without considering potential changes over time. Finally, the study was limited to DFU patients over the age of 40 in the American population, and caution should be exercised when extrapolating these findings to other populations. To clarify the predictive value of elevated BUN for DFU, as well as whether there is a bidirectional causal relationship between the 2, future clinical studies with larger sample sizes and more detailed stratification of covariates are needed.

## 5. Conclusion

Overall, high BUN levels are associated with an increased risk of developing DFU. Diabetic patients with high BUN levels should be screened early and actively prevented from developing DFU. These findings require further prospective studies to provide more evidence.

## Author contributions

**Data curation:** Ruijing Li, Changfan Li.

**Formal analysis:** Ruijing Li, Changfan Li.

**Methodology:** Ruijing Li.

**Resources:** Ruijing Li.

**Supervision:** Changfan Li.

**Writing – original draft:** Ruijing Li.

**Writing – review & editing:** Changfan Li.
